# Effects of Acidic Media on the Mechanical and Optical Properties of Digitally Designed Restorative Materials

**DOI:** 10.1111/jerd.70197

**Published:** 2026-05-26

**Authors:** Ismini Papoulidou, Petros Mourouzis, Andrea Baldi, Nicola Scotti, Dimitrios Dionysopoulos

**Affiliations:** ^1^ Department of Operative Dentistry, School of Dentistry Aristotle University of Thessaloniki Thessaloniki Greece; ^2^ Department of Surgical Sciences, Dental School University of Turin Turin Italy

**Keywords:** 3D‐printed resin, beverage, CAD/CAM restorative materials, color, erosion, gastric acid, surface hardness, surface roughness, translucency

## Abstract

**Objective:**

This investigation assessed how acidic environments affect the optical behavior, surface roughness, and hardness of a newly developed 3D‐printed permanent resin compared with commonly used computer‐aided design/computer‐aided manufacturing restorative blocks.

**Materials and Methods:**

The materials tested were lithium disilicate, a polymer‐infiltrated ceramic network, a nanoceramic resin block, and a 3D‐printed composite. Specimens were stored in hydrochloric acid and Coca‐Cola, with distilled water as the control medium. Changes in color, translucency, roughness, and hardness were measured before and after immersion. Data were analyzed using R (v4.5) with a significance level of *p* < 0.05.

**Results:**

Lithium disilicate showed the greatest resistance to acidic conditions in both optical and mechanical properties. The polymer‐infiltrated ceramic network and filler press and monomer infiltration materials showed intermediate stability under acidic challenges across all measured parameters. The 3D‐printed resin was the least stable material. Gastric acid caused more pronounced deterioration than Coca‐Cola, while water produced minimal changes.

**Conclusions:**

Acidic exposure negatively affected the performance of all tested restorative materials. Lithium disilicate remained largely unaffected, whereas the 3D‐printed permanent resin showed significant vulnerability. These findings emphasize that material choice is critical for patients prone to erosive challenges such as reflux or frequent soft drink intake.

## 1 Introduction

Computer‐aided design and computer‐aided manufacturing (CAD/CAM) technology in dentistry has grown substantially, and the use of design software and intraoral scanners has increased the number of restorations made chairside in a single visit [1]. A wide range of materials is currently available for CAD/CAM restorations, broadening the available options for different clinical demands [2, 3]. Lithium disilicate glass‐ceramics remain among the most commonly used, and a fully crystallized material was recently introduced featuring patented high‐density micronization technology designed for use without a firing step [4]. Resin‐matrix ceramics, or polymer‐infiltrated ceramic network (PICN) materials, combine a ceramic framework with an infiltrated polymer phase [5]. Polymer materials used in CAD/CAM technology are produced using advanced polymerization strategies such as high temperature/pressure processing, filler press techniques, and monomer infiltration [5, 6, 7, 8, 9]. Materials manufactured by the filler press and monomer infiltration (FPMI) method have been reported to exhibit improved mechanical and chemical properties [9, 10].

Chairside restorations have relied primarily on subtractive manufacturing, whereas additive manufacturing [11, 12, 13] has recently been introduced for prosthetic applications, including crowns, dentures, and splints [14, 15]. Advances in 3D printing have enabled ceramic‐filled resins intended to enhance esthetics, color durability, and mechanical performance, suggesting potential for definitive restorations [16, 17, 18]. New 3D‐printed resin materials have evolved to contain up to 30%–50% glass fillers by weight [19] and have been investigated for the fabrication of single crowns and other indirect restorations [20]. Endogenous and dietary acids can progressively damage dental hard tissues [21]. Gastric juice is a hydrochloric acid–based endogenous fluid with a very low pH (≈0.9–1.5) that may reach the oral cavity during vomiting or gastroesophageal reflux and contribute to degradation of dental tissues and restorative materials [22, 23]. Among exogenous acids, Coca‐Cola is frequently used in laboratory studies because of its widespread consumption and erosive potential [22, 24]. However, the absence of standardized erosion protocols results in variable outcomes, with studies using either cyclic or continuous exposure models [25, 26, 27]. Considering the growing use of chairside materials for indirect restorations, it is important to evaluate the effect of acidic exposure on milled and 3D‐printed materials. Therefore, this study evaluated the effects of acidic immersion on the optical behavior, surface characteristics, and mechanical properties of different CAD/CAM restorative materials and a 3D‐printed permanent resin. The research hypothesis was that material‐dependent differences would be detected within the same immersion solution and that the immersion solution would affect the magnitude of these changes.

## 2 Materials and Methods

### 2.1 Study Design

The tested materials comprised three CAD/CAM blocks—lithium disilicate, a polymer‐infiltrated ceramic network material, and a filler‐pressed/monomer‐infiltrated nanoceramic—and one 3D‐printed resin.

### 2.2 Sample Preparation

Eighty rectangular specimens (14 × 12 × 1.5 mm) were assigned to four material groups (*n* = 20). Sixty specimens were sectioned from CAD/CAM blocks using a 0.3‐mm diamond‐coated low‐speed precision saw (IsoMet 1000; Buehler, Lake Bluff, IL, USA) under water irrigation. Surfaces were flattened and polished under water cooling with silicon carbide papers (P280, P600, P1200, P2400, and P4000) at 150 rpm (TG 250; Jean Wirtz, Düsseldorf, Germany), and thickness was verified with a digital micrometer (Digimatic Micrometer Series 203 MDC‐MX Lite; Mitutoyo, Kawasaki, Japan).

Twenty specimens were fabricated from a permanent 3D‐printing resin (Permanent Crown; Formlabs, Somerville, MA, USA) using a stereolithography printer (Form 3B+; Formlabs, Somerville, MA, USA). Specimens were digitally designed, exported as STL files, and processed in PreForm software (Formlabs, Somerville, MA, USA). Printing was performed using the manufacturer's preset 50 μm settings [28], with a 90° build orientation. After printing, specimens were washed in 99% isopropyl alcohol for 3 min using an automated washing unit (Form Wash; Formlabs, Somerville, MA, USA), air‐dried, and post‐cured (Form Cure; Formlabs, Somerville, MA, USA) for 20 min at 60°C with supports attached [28]. After support removal, a second post‐curing cycle was performed for 20 min at 60°C [29]. Surface residues were removed by sandblasting with 50 μm aluminum oxide at 2 bar, and the surface closest to the build platform was polished [29] using the same protocol applied to the CAD/CAM materials.

All specimens were ultrasonically cleaned in distilled water for 10 min, air‐dried, and stored in the dark at room temperature (23°C ± 1°C) until testing. Material compositions (wt%) were obtained from manufacturer specifications (Table 1).

**TABLE 1:** Description of materials according to the manufacturer.

Material	Material type	Composition	Shade/LOT number	Manufacturer
Initial LiSi Block	Lithium disilicate	55%–80% SiO_2_, 10%–30% Li_2_O; 5%–20% other oxides; pigments: trace	A2/2205211	GC Corporation, Tokyo, Japan
Vita Enamic	PICN, HT/HP	Organic matrix: UDMA, TEGDMA (14 wt%; 25 vol%). Inorganic filler: feldspar ceramic enriched with aluminum oxide (75 vol%; 86 wt%).	A2/60450	VITA Zahnfabrik H. Rauter KG, Bad Säckingen, Germany
Katana Avencia	Nanoceramic, FPMI	Organic matrix: UDMA, TEGDMA. Inorganic filler: SiO_2_ (60 nm), Al_2_O_3_ (20 nm) (62 wt%).	A2/0004488	Kuraray Noritake Dental, Tokyo, Japan
Formlabs Permanent Crown	Methacrylic acid ester‐based resin	Organic matrix: ≥ 50–< 75 wt% Bis‐EMA. Inorganic filler: silanized dental glass (30–50 wt%).	A2/20240216	Formlabs, Somerville, MA, USA

Abbreviations: Bis‐EMA, bisphenol A ethoxylate dimethacrylate; FPMI, filler press and monomer infiltration; HT/HP, high temperature/high pressure; PICN, polymer‐infiltrated ceramic network; UDMA, urethane dimethacrylate; TEGDMA, triethylene glycol dimethacrylate.

### 2.3 Erosive Media

Baseline measurements were obtained for all specimens before exposure. Specimens were randomly allocated to three storage media: (a) laboratory‐prepared simulated gastric acid (5% HCl, adjusted to pH = 2) [23], (b) a phosphoric acid–containing carbonated beverage (Coca‐Cola, pH = 2.5), and (c) distilled water as the control group (pH = 7).

Each material group (*n* = 20) was immersed in gastric acid (*n* = 8), Coca‐Cola (*n* = 8), or water (*n* = 4). Specimens were immersed in 10 mL of solution in sealed glass containers, and pH was verified before immersion using a calibrated pH meter (mPA‐210P; MS Tecnopon, Piracicaba, Brazil).

**FIGURE 1:**
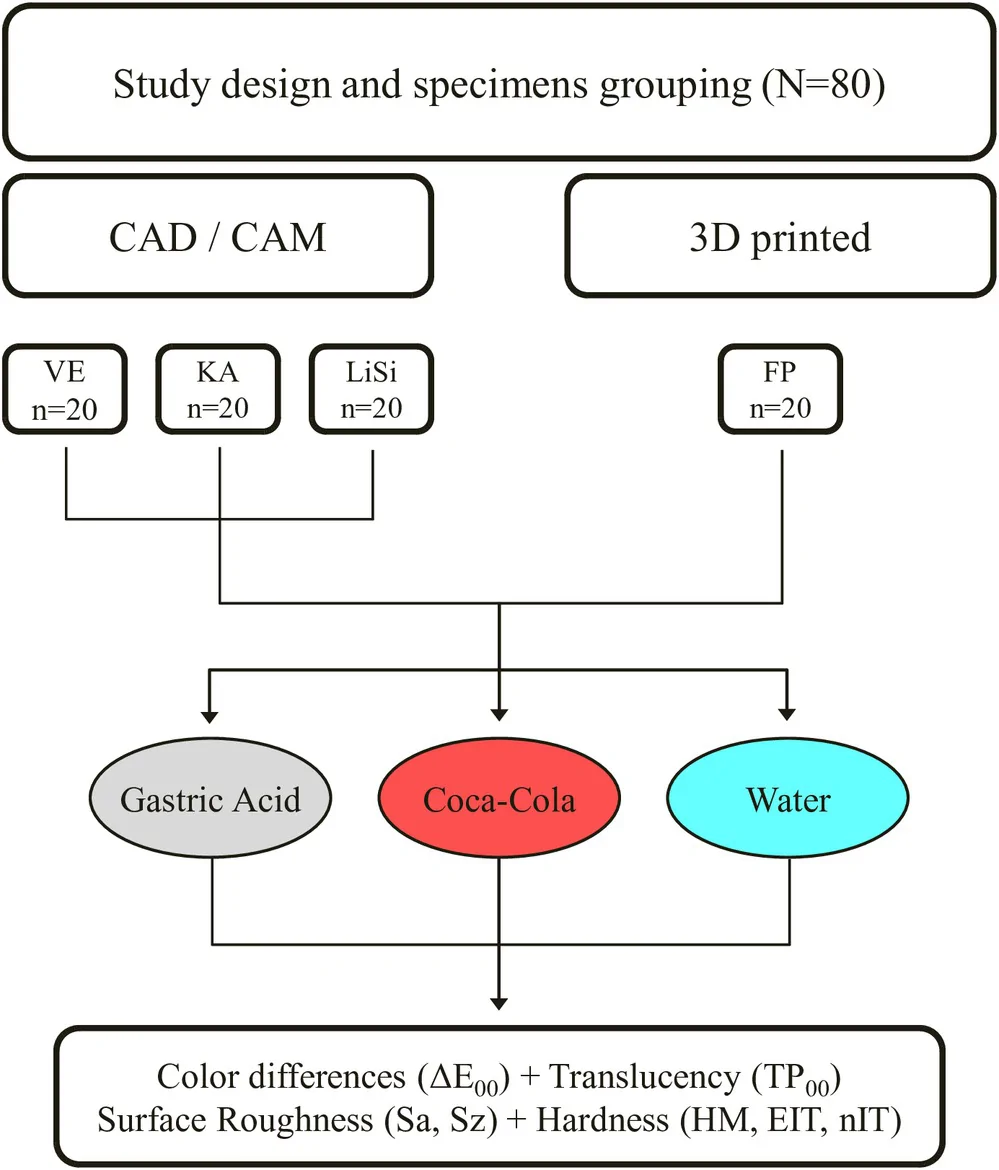
Study design and sample grouping. Specimens from Vita Enamic (VE), Katana Avencia (KA), Initial LiSi Block (LiSi), and Formlabs Permanent Crown (FP) were allocated to gastric acid, Coca‐Cola, and distilled water.

All specimens were stored in an incubator (Thermo Fisher Scientific) at 37°C for 91 h. The immersion time under acidic conditions corresponded to 12 months of clinical exposure, assuming daily exposure of approximately 15 min [23]. This estimate was based on the premise that a bulimic individual purges three times daily, with each episode lasting about 5 min. Specimens were immersed in Coca‐Cola for the same duration as in gastric acid [22]. After immersion, specimens were ultrasonically cleaned in distilled water for 10 min and air‐dried (Figure 1).

### 2.4 Measurement of Surface Roughness

Surface roughness was assessed using a three‐dimensional optical non‐contact profilometer (Contour GT; Bruker, Tucson, AZ, USA) with Vision64 software. Each specimen was measured in three distinct non‐overlapping areas over a scan area of approximately 632 × 472 μm. The first measurement was obtained at the center of the specimen, and the other two at randomly selected peripheral locations. The areal roughness parameters Sa (arithmetical mean height of the surface) and Sz (maximum height of the surface) were recorded in μm. Surface roughness findings were interpreted in relation to the 0.2 μm threshold reported for bacterial plaque retention [30]. Surface topography maps were generated by the profilometer software from the scan data and were presented as representative qualitative images to visualize surface morphology.

### 2.5 Measurements of Optical Properties

Initial and post‐exposure optical measurements were obtained using a UV–VIS spectrophotometer (UV‐2401PC; Shimadzu, Kyoto, Japan) with UVPROBE software (v2.21; Shimadzu). To ensure consistent specimen positioning, a custom black‐and‐white holder was fabricated using a 3D printer. The instrument was calibrated with standard pressed barium sulfate according to the manufacturer's instructions. Measurements were performed in reflectance mode using the D65 illuminant and 2° standard observer, with an ISR‐240A integrating sphere and a 7‐mm aperture, on black and white backgrounds. A drop of glycerol (refractive index, *n* = 1.472) was applied between the specimen and the background to reduce optical discontinuities. Each specimen was measured three consecutive times, and the mean L*, a*, and b* values were recorded.

Translucency (TP_00_) was calculated using the CIEDE2000 color difference formula (1:1:1): [31]

$$TP_{00}={\left[{\left(\frac{\Delta L_{\mathrm{BW}}^{\prime }}{K_{L}S_{L}}\right)}^{2}+{\left(\frac{\Delta C_{\mathrm{BW}}^{\prime }}{K_{C}S_{C}}\right)}^{2}+{\left(\frac{\Delta H_{\mathrm{BW}}^{\prime }}{K_{H}S_{H}}\right)}^{2}+R_{T}\left(\frac{\Delta C_{\mathrm{BW}}^{\prime }}{K_{C}S_{C}}\right)\left(\frac{\Delta H_{\mathrm{BW}}^{\prime }}{K_{H}S_{H}}\right)\right]}^{1/2}$$

where subscripts *B* and *W* refer to measurements over black and white backgrounds, respectively; $\Delta L_{\mathrm{BW}}^{\prime }$, $\Delta C_{\mathrm{BW}}^{\prime }$, and $\Delta H_{\mathrm{BW}}^{\prime }$ are the modified differences in lightness, chroma, and hue between the two backgrounds; $R_{T}$ is the rotation function describing the interaction between chroma and hue differences in the blue region; $S_{L}$, $S_{C}$, and $S_{H}$ are weighting functions; and $K_{L}$, $K_{C}$, and $K_{H}$ are parametric correction factors.

Color change (Δ*E*_00_) was calculated between baseline and post‐exposure measurements using the CIEDE2000 formula: [32]

$$\Delta E_{00}={\left[{\left(\frac{\Delta L^{\prime }}{K_{L}S_{L}}\right)}^{2}+{\left(\frac{\Delta C^{\prime }}{K_{C}S_{C}}\right)}^{2}+{\left(\frac{\Delta H^{\prime }}{K_{H}S_{H}}\right)}^{2}+R_{T}\left(\frac{\Delta C^{\prime }}{K_{C}S_{C}}\right)\left(\frac{\Delta H^{\prime }}{K_{H}S_{H}}\right)\right]}^{1/2}$$

where $\Delta L^{\prime }$ is the change in modified lightness, $\Delta C^{\prime }$ is the difference in modified chroma, and $\Delta H^{\prime }$ is the difference in modified hue, while $R_{T}$, $S_{L}$, $S_{C}$, and $S_{H}$ are weighting factors. The parametric factors $K_{L}$, $K_{C}$, and $K_{H}$ were set to 1.

Color changes were considered perceptible when ΔE_00_ > 0.8 and clinically acceptable when Δ*E*_00_ < 1.8 [33]. Translucency changes were considered perceptible when ΔTP_00_ > 0.62 and clinically acceptable when ΔTP_00_ < 2.62 [31]. Color measurements were performed with the guidance of ISO/TR 28642:2016 for instrumental dental color assessment and interpretation of color differences [34].

### 2.6 Measurement of Surface Hardness

Surface hardness was assessed using a nanoindentation tester (HIT 300; Anton Paar TriTec SA, Corcelles‐Cormondrèche, Switzerland). Calibration indents were performed on fused silica (elastic modulus: 71.3 GPa; hardness: 8.9 GPa). A Berkovich diamond indenter was used with a maximum load of 20 mN, a 30 s loading time to maximum load, a 15 s hold period, an approach speed of 4000 nm/min, and an acquisition rate of 10 Hz under quadratic loading. Poisson's ratio was assumed to be 0.3 for all tested materials [7]. Six indentations were performed on the upper surface of each specimen, spaced 100 μm apart, at room temperature (23°C ± 1°C). Force–displacement data were analyzed using Indentation Software Version 10.2.4. Mean values for Martens hardness (HM, N/mm^2^), indentation modulus (EIT, GPa), and elastic index (nIT, %), defined as the ratio of elastic deformation energy to total energy, were calculated. Indentation modulus was determined according to instrumented indentation analysis based on the Oliver–Pharr method [35].

### 2.7 Scanning Electron Microscopy With Energy‐Dispersive X‐Ray Spectroscopy (SEM–EDS)

Specimen surfaces were examined using a scanning electron microscope (JEOL, JSM‐840, Tokyo, Japan). Elemental analysis was performed by energy‐dispersive x‐ray spectroscopy (EDS). Specimens were sputter‐coated with carbon to a thickness of approximately 200 Å using a low‐pressure vacuum evaporator and examined at an accelerating voltage of 20 kV. Secondary electron images (SEI) were obtained from the top surfaces at 100×, 500×, and 2000× magnification, with a 10 μm scale bar, to evaluate morphological differences among the experimental groups.

### 2.8 Statistical Analysis

Sample size was determined a priori using power analysis based on pilot data, assuming a significance level of *α* = 0.05 and power (1 − *β*) of 0.80, with effect size estimated from preliminary measurements. Calculations were performed using G*Power software (version 3.1.9.2; Heinrich‐Heine‐Universität Düsseldorf, Germany) for Mac.

All analyses were performed using R (v4.5), with statistical significance set at *p* < 0.05.

For roughness outcomes (ΔSa and ΔSz), optical outcomes (Δ*E*₀₀ and ΔTP₀₀), and nanoindentation outcomes (ΔHM, ΔEIT, and ΔnIT), two‐way linear models including material, solution, and their interaction as fixed effects were used (outcome ~ material * solution). ΔTP₀₀, ΔSa, ΔSz, ΔHM, ΔEIT, and ΔnIT were calculated as paired changes after immersion (After–Before), whereas Δ*E*₀₀ was expressed as the CIEDE2000 color difference. Because the design was unbalanced for some outcomes and residual diagnostics indicated heteroskedasticity and/or non‐normal residual behavior, omnibus tests were based on Type III tests using HC3 heteroskedasticity‐consistent covariance estimation. For significant material × solution interactions, estimated marginal means were compared within each solution using Holm‐adjusted post hoc tests, and simple solution effects were examined within each material.

Distilled water served as a non‐erosive control to verify measurement stability and isolate the effects of acidic exposure. Based on pilot data, variability in distilled water was lower than in acidic media; therefore, a smaller sample size (*n* = 4 per material) was considered sufficient for control comparisons, whereas larger groups (*n* = 8) were used for acidic conditions due to greater variability and expected effect sizes.

## 3 Results

Acidic media produced greater changes than distilled water, with the magnitude of these changes varying among materials. Results are reported as mean ± standard deviation, with compact letter displays denoting significant within‐solution differences at *p* < 0.05 in Tables 2, 3, 4.

**TABLE 2:** Changes in surface roughness after immersion.

Material	Medium	ΔSa (μm)	ΔSz (μm)
LiSi	Gastric acid	0.01 ± 0.01^a^	0.66 ± 0.28^a^
Vita Enamic	Gastric acid	0.04 ± 0.01^b^	1.75 ± 0.03^b^
Katana Avencia	Gastric acid	0.02 ± 0.01^c^	1.35 ± 0.38^c^
3D‐printed resin	Gastric acid	0.07 ± 0.01^d^	2.67 ± 0.18^d^
LiSi	Coca‐Cola	0.01 ± 0.01^a^	0.63 ± 0.13^a^
Vita Enamic	Coca‐Cola	0.03 ± 0.01^b^	1.66 ± 0.39^b^
Katana Avencia	Coca‐Cola	0.01 ± 0.01^a^	1.23 ± 0.20^c^
3D‐printed resin	Coca‐Cola	0.05 ± 0.01^c^	2.38 ± 0.21^d^
LiSi	Distilled water	0.00 ± 0.01^a^	0.05 ± 0.01^a^
Vita Enamic	Distilled water	0.00 ± 0.01^a^	0.05 ± 0.02^a^
Katana Avencia	Distilled water	0.00 ± 0.01^a^	0.05 ± 0.01^a^
3D‐printed resin	Distilled water	0.01 ± 0.01^a^	0.07 ± 0.02^a^

*Note:* Values are presented as mean ± standard deviation. Different superscript lowercase letters within the same outcome and storage medium indicate statistically significant differences between materials based on Holm‐adjusted estimated marginal mean comparisons (*p* < 0.05).

**TABLE 3:** Changes in optical properties after immersion.

Material	Δ*E*_00_ Gastric acid	Δ*E*_00_ Cssssoca‐Cola	Δ*E*_00_ Distilled water	ΔTP_00_ Gastric acid	ΔTP_00_ Coca‐Cola	ΔTP_00_ Distilled water
LiSi	1.18 ± 0.03^a^	1.13 ± 0.02^a^	0.14 ± 0.10^a^	−0.43 ± 0.20^a^	−0.33 ± 0.22^a^	−0.08 ± 0.06^a^
Vita Enamic	1.49 ± 0.13^b^	1.33 ± 0.06^b^	0.14 ± 0.05^a^	−1.91 ± 0.63^b^	−1.23 ± 0.57^b^	−0.04 ± 0.01^a^
Katana Avencia	1.64 ± 0.05^c^	1.47 ± 0.04^c^	0.24 ± 0.07^a^	−0.90 ± 0.45^c^	−0.70 ± 0.33^c^	−0.07 ± 0.03^a^
3D‐printed resin	1.76 ± 0.06^d^	1.62 ± 0.04^d^	0.26 ± 0.08^a^	−3.04 ± 0.91^d^	−2.16 ± 0.75^d^	−0.14 ± 0.08^a^

*Note:* Values are presented as mean ± standard deviation. Different superscript lowercase letters within the same outcome and storage medium indicate statistically significant differences between materials based on Holm‐adjusted estimated marginal mean comparisons (*p* < 0.05).

**TABLE 4:** Changes in nano‐mechanical properties after immersion.

Material	Medium	ΔHM (N/mm^2^)	ΔEIT (GPa)	ΔnIT (%)
LiSi	Gastric acid	−0.04 ± 0.02^a^	−0.86 ± 0.40^a^	−0.27 ± 0.19^a^
Vita Enamic	Gastric acid	−0.07 ± 0.01^b^	−1.75 ± 0.34^b^	−1.46 ± 0.77^b^
Katana Avencia	Gastric acid	−0.06 ± 0.02^b^	−1.48 ± 0.52^b^	−0.64 ± 0.29^c^
3D‐printed resin	Gastric acid	−0.13 ± 0.03^c^	−4.15 ± 0.36^c^	−2.40 ± 0.33^d^
LiSi	Coca‐Cola	−0.02 ± 0.01^a^	−0.63 ± 0.37^a^	−0.19 ± 0.10^a^
Vita Enamic	Coca‐Cola	−0.06 ± 0.02^b^	−1.57 ± 0.56^b^	−0.95 ± 0.21^b^
Katana Avencia	Coca‐Cola	−0.05 ± 0.02^b^	−1.22 ± 0.63^b^	−0.43 ± 0.15^c^
3D‐printed resin	Coca‐Cola	−0.11 ± 0.01^c^	−3.36 ± 0.88^c^	−1.59 ± 0.49^d^
LiSi	Distilled water	−0.01 ± 0.01^a^	−0.04 ± 0.02^a^	−0.02 ± 0.01^a^
Vita Enamic	Distilled water	−0.01 ± 0.01^a^	−0.03 ± 0.02^a^	−0.01 ± 0.01^a^
Katana Avencia	Distilled water	0.00 ± 0.01^a^	−0.02 ± 0.01^a^	−0.02 ± 0.03^a^
3D‐printed resin	Distilled water	−0.01 ± 0.01^a^	−0.03 ± 0.02^a^	−0.02 ± 0.01^a^

*Note:* Values are presented as mean ± standard deviation. Different superscript lowercase letters within the same outcome and storage medium indicate statistically significant differences between materials based on Holm‐adjusted estimated marginal mean comparisons (*p* < 0.05).

### 3.1 Surface Roughness (Sa and Sz)

For ΔSa and ΔSz, significant effects were found for material (F[3, 68] = 51.78 and 101.06, respectively; *p* < 0.001), storage medium (F[2, 68] = 3.92, *p* = 0.024 and F[2, 68] = 82.89, *p* < 0.001, respectively), and the material × storage medium interaction (F[6, 68] = 6.70 and 103.27, respectively; *p* < 0.001). Because the interactions were significant, interpretation focused on simple effects.

Material differences were mainly observed in gastric acid and Coca‐Cola for ΔSa and ΔSz. Water produced no significant between‐material differences for either ΔSa or ΔSz.

**FIGURE 2:**
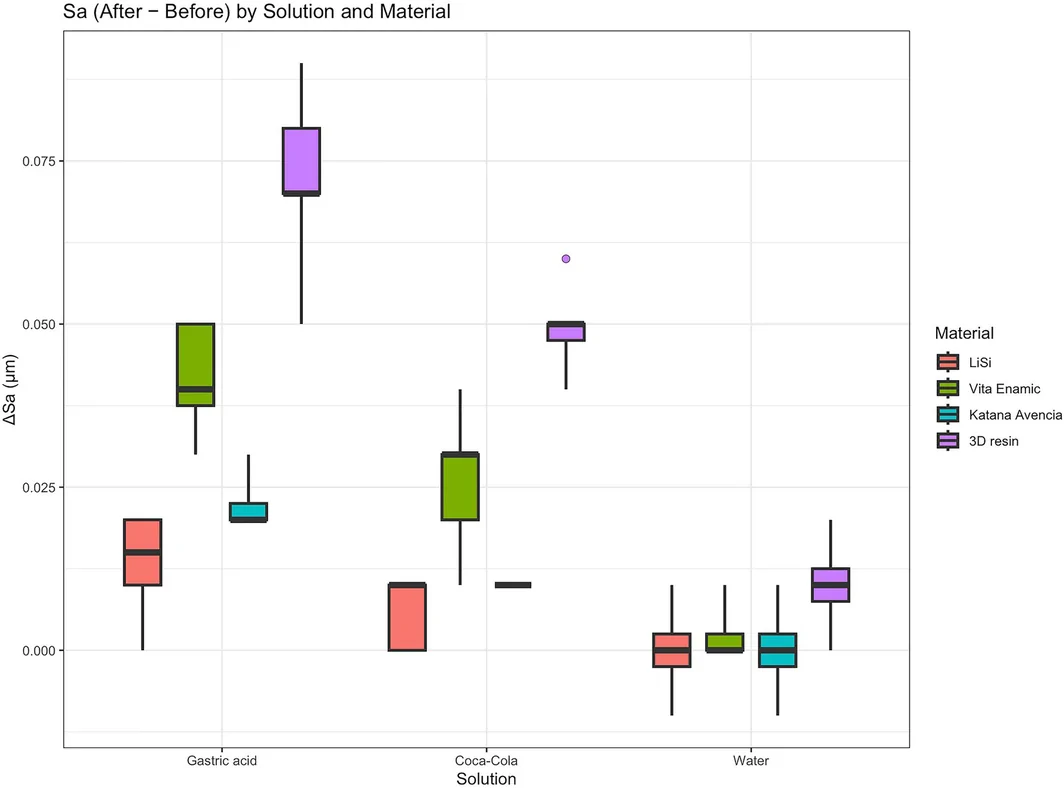
Boxplot of the change in average surface roughness (ΔSa; After–Before, μm) for each material following immersion in gastric acid, Coca‐Cola, and water.

For ΔSa, in gastric acid, all materials differed significantly, in the order 3D‐printed resin > Vita Enamic > Katana Avencia > LiSi. In Coca‐Cola, the same trend was observed, except that Katana Avencia and LiSi did not differ significantly (Holm *p* = 0.059) (Figure 2).

For ΔSz, all materials differed significantly in both gastric acid and Coca‐Cola, following the order 3D‐printed resin > Vita Enamic > Katana Avencia > LiSi.

### 3.2 Optical Properties (ΔE₀₀ and ΔTP₀₀)

For ΔE₀₀ and ΔTP₀₀, significant effects were found for material (F[3,68] = 277.02 and 28.71, respectively; *p* < 0.001), solution (F[2, 68] = 149.31 and 10.68, respectively; *p* < 0.001), and the material × solution interaction (F[6, 68] = 9.40 and 20.23, respectively; *p* < 0.001). Because the interactions were significant, interpretation focused on simple effects. Solution significantly affected both outcomes for all materials (all *p* < 0.001), whereas no significant differences between materials were detected in water (Holm *p* ≥ 0.05). Within the acidic media, a clear ranking was observed: for ΔE₀₀, 3D‐printed resin > Katana Avencia > Vita Enamic > LiSi, whereas for ΔTP₀₀, 3D‐printed resin > Vita Enamic > Katana Avencia > LiSi.

### 3.3 Surface Hardness (HM, EIT, nIT)

**FIGURE 3:**
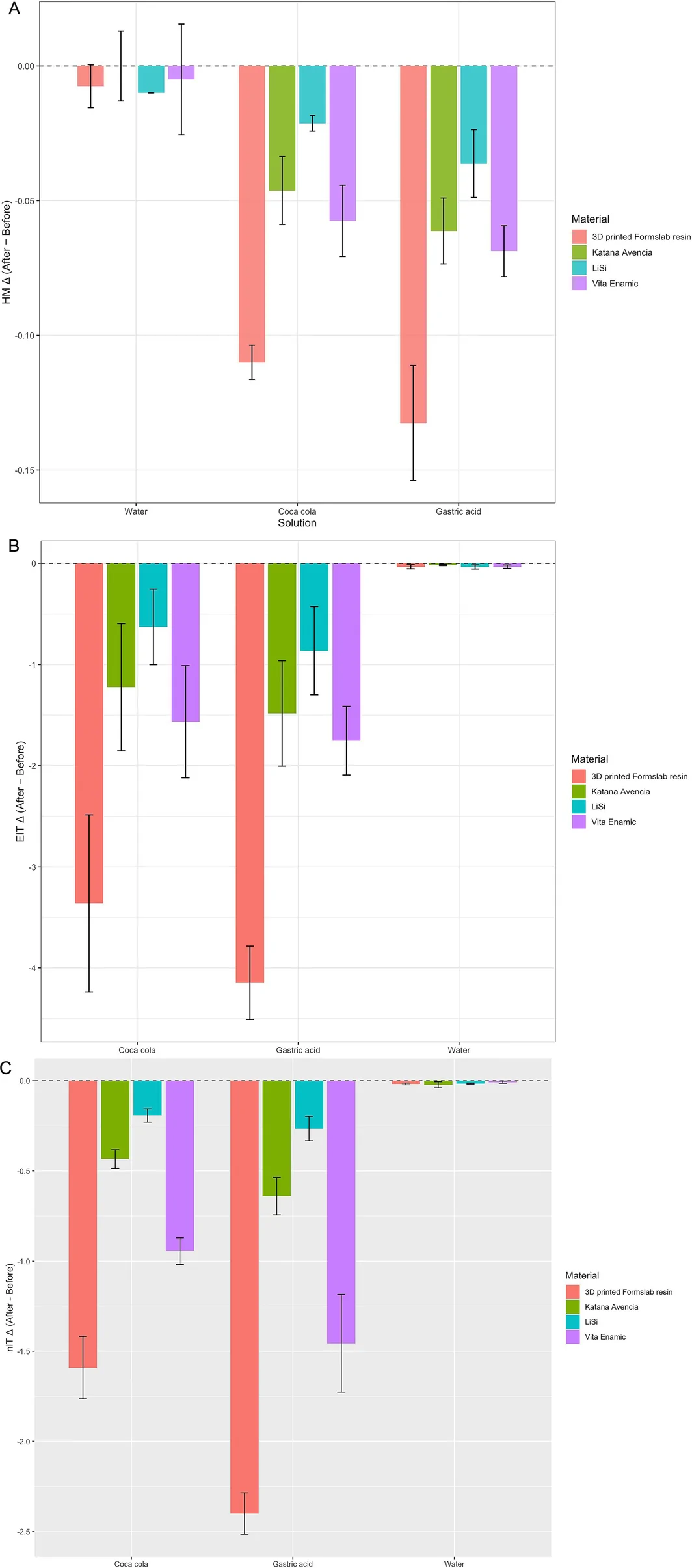
Mean changes in (A) Martens hardness (ΔHM, N/mm^2^), (B) indentation modulus (ΔEIT, GPa), and (C) elastic index (ΔnIT, %) after immersion in gastric acid, Coca‐Cola, or distilled water. Values represent After–Before changes. Error bars indicate standard deviation. Different lowercase letters indicate significant differences between materials within the same medium and outcome (Holm‐adjusted post hoc comparisons, *p* < 0.05).

For HM, EIT, and nIT, significant effects were found for material (F[3, 68] = 78.53, 69.62, and 82.56, respectively; *p* < 0.001), solution (F[2, 68] = 200.78, 421.09, and 198.89, respectively; *p* < 0.001), and the material × solution interaction (F[6, 68] = 84.27, 58.62, and 58.21, respectively; *p* < 0.001). The significant interaction indicates that the effect of immersion solution differed among materials. No significant inter‐material differences were detected in water for any parameter. For parameters HM and EIT, the order was 3D‐printed resin > Vita Enamic ≈ Katana Avencia > LiSi, whereas for nIT the order was 3D‐printed resin > Vita Enamic > Katana Avencia > LiSi (Figure 3).

### 3.4 SEM–EDS Observations

SEM images (100×, 500× and 2000×) revealed material‐dependent responses to storage media. Lithium disilicate remained relatively smooth in water but developed scattered pores and irregularities after acid exposure, with gastric acid producing deeper and more heterogeneous surface alterations than Coca‐Cola. PICN exhibited pronounced porosity in gastric acid, while changes after Coca‐Cola were limited and localized. FPMI material maintained a relatively smooth surface under all conditions, showing only minor topographical features. In contrast, the 3D‐printed resin exhibited surface alterations and showed porosity and heterogeneity following acidic immersion.

Qualitative evaluation of the EDS wt% values suggested material‐dependent surface compositional changes after acidic exposure. FPMI and lithium disilicate showed more evident relative shifts toward Si‐rich surface profiles, which may reflect selective dissolution or depletion of minor glass/ceramic constituents. PICN showed only limited elemental variation, indicating a comparatively stable surface inorganic composition. The 3D‐printed resin showed increased relative Si and decreased Ba, more pronounced after gastric acid than after Coca‐Cola exposure, which may indicate filler‐related surface alteration.

**FIGURE 4:**
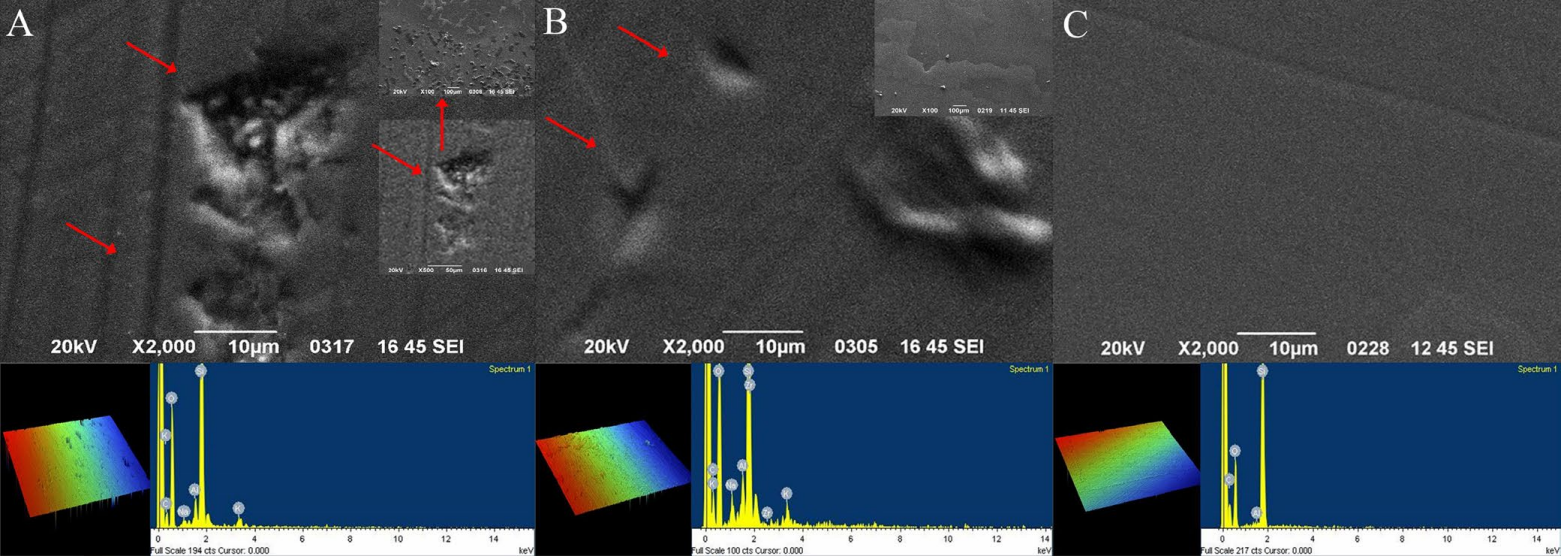
Representative SEM micrographs, surface topography maps, and EDS spectra of lithium disilicate (LiSi) after exposure to different media: (A) gastric acid, (B) Coca‐Cola, and (C) distilled water (control). (A) SEM image of lithium disilicate (LiSi) after gastric acid exposure (2000×, 500×, and 100×), showing a localized degraded region with micro‐pits and irregular depressions. Parallel polishing grooves remain evident, with superimposed pits and depressions indicating acid‐induced surface degradation (arrows). (B) SEM image of lithium disilicate (LiSi) after Coca‐Cola exposure (2000× and 100×), showing localized pits and shallow depressions (arrows). (C) SEM image of lithium disilicate (LiSi) in distilled water (control) at 2000× showing a relatively smooth and homogeneous surface without visible defects.

**FIGURE 5:**
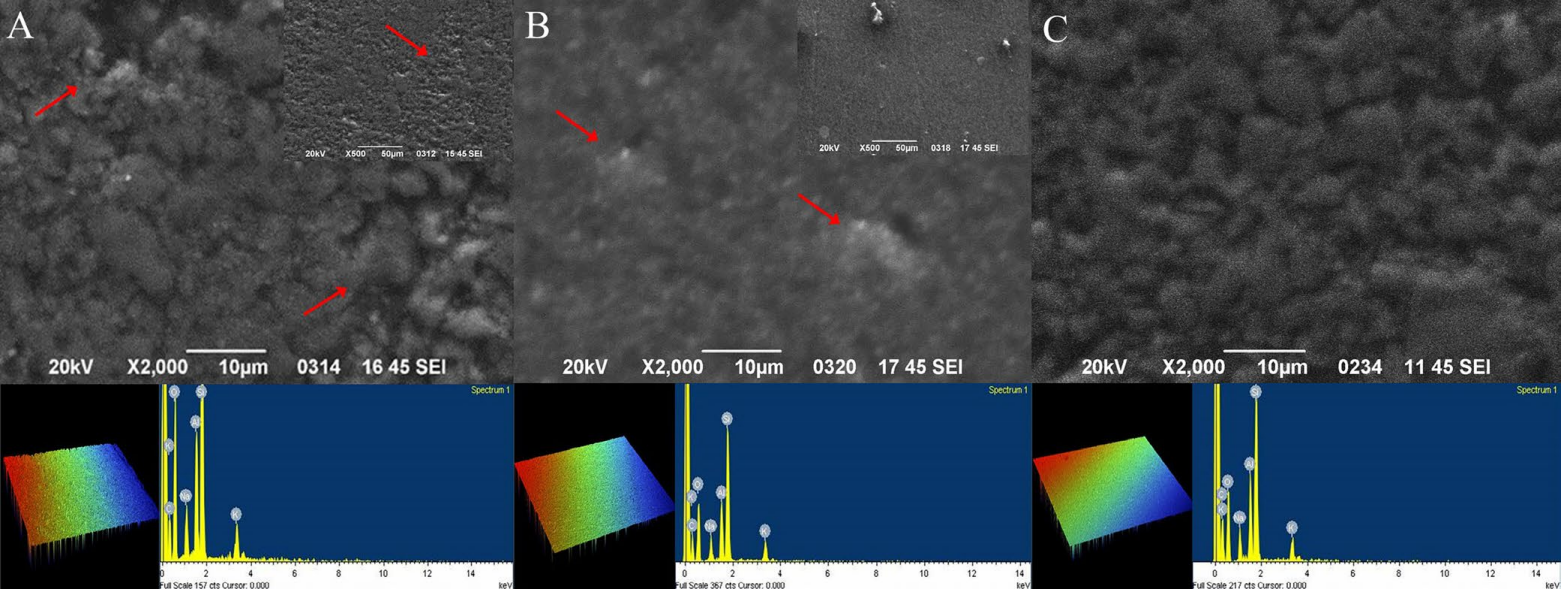
SEM micrographs, surface maps, and EDS spectra of Vita Enamic after exposure to different media: (A) gastric acid, (B) Coca‐Cola, and (C) distilled water (control). (A) SEM image of Vita Enamic after gastric acid exposure (2000× and 500×), showing numerous localized surface irregularities (arrows). (B) SEM image of Vita Enamic after Coca‐Cola exposure (2000× and 500×), showing small protrusions and shallow surface irregularities (arrows). (C) SEM image of Vita Enamic in distilled water (control) at 2000× showing a relatively smooth and homogeneous surface morphology.

**FIGURE 6:**
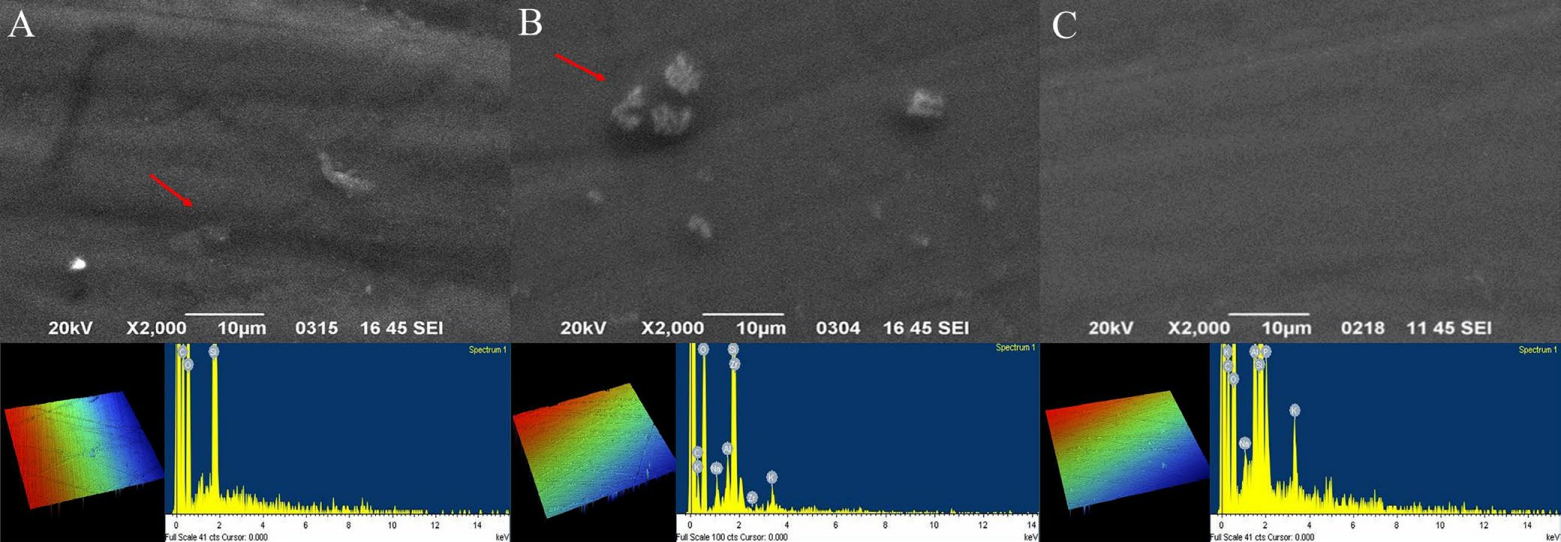
SEM micrographs, surface maps, and EDS spectra of Katana Avencia after exposure to different media: (A) gastric acid, (B) Coca‐Cola, and (C) distilled water (control). (A) SEM image of Katana Avencia after gastric acid exposure (2000×) showing minor localized surface irregularities (arrow) without large pits or clusters. (B) SEM image of Katana Avencia after Coca‐Cola exposure (2000×) showing clustered protruding particles (arrow), while the surrounding surface remains relatively smooth. (C) SEM image of Katana Avencia in distilled water (control) at 2000× showing a relatively smooth and homogeneous surface morphology.

**FIGURE 7:**
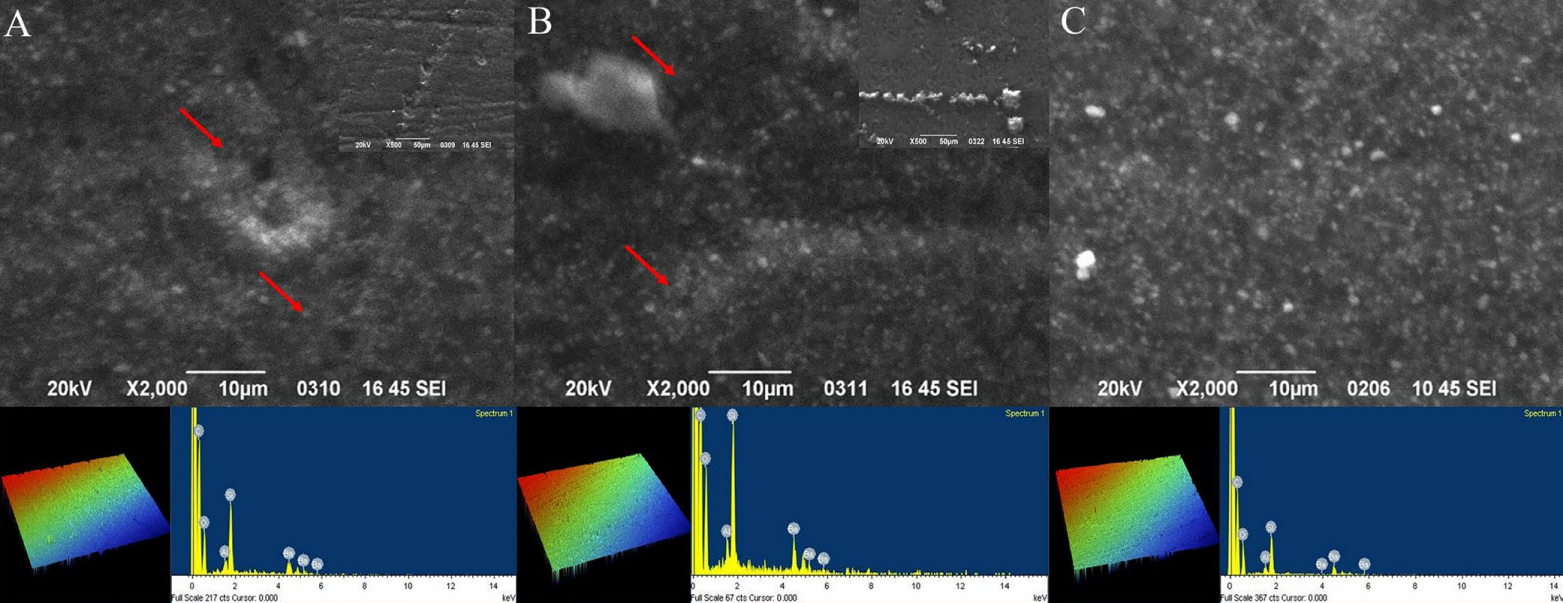
SEM micrographs, surface maps, and EDS spectra of 3D‐printed Formlabs Permanent Crown resin after exposure to different media: (A) gastric acid, (B) Coca‐Cola, and (C) distilled water (control). (A) SEM image of the 3D‐printed resin after gastric acid exposure (2000× and 500×) showing localized irregularities and small pits (arrows). Polishing grooves are partially disrupted by pits and surface degradation, indicating erosive effects (500×). (B) SEM image of the 3D‐printed resin after Coca‐Cola exposure (2000× and 500×) showing protruding particles and localized surface irregularities (arrows). (C) SEM image of the 3D‐printed resin in distilled water (control) at 2000× showing a relatively uniform surface with dispersed small particles.

Representative SEM micrographs, surface topography maps, and EDS spectra for all materials and storage conditions are presented in Figures 4, 5, 6, 7.

## 4 Discussion

The present findings revealed distinct material‐dependent degradation patterns under acidic conditions. The 3D‐printed resin was the most susceptible to degradation, whereas lithium disilicate was the most stable. PICN and FPMI showed a parameter‐dependent response: PICN exhibited greater roughness increase, translucency loss, and elastic index change, whereas FPMI showed greater discoloration. Both materials behaved similarly for Martens hardness and indentation modulus.

### 4.1 Surface Roughness

All materials remained below the 0.2 μm surface roughness threshold reported for bacterial plaque retention, except the 3D‐printed resin after immersion in gastric acid and Coca‐Cola [30]. Studies report inconsistent findings regarding the effect of acidic media on surface roughness, with both increases and decreases described, likely due to differences in immersion protocols, polishing procedures, and simulation models [22, 23, 25, 26]. Previous studies have shown that lithium disilicate exhibits complex, time‐dependent surface responses to acid. Dissolution of the glassy matrix initially exposes crystalline grains, increasing roughness, whereas prolonged exposure can result in smoothing as crystals are gradually removed, revealing the glass matrix [26]. In the present study, lithium disilicate maintained the lowest overall roughness changes among all materials. SEM observations revealed nanometric pores, while profilometry indicated only minor alterations, a discrepancy also reported previously [26]. PICN materials demonstrated significant increases in both Sa and Sz after gastric acid and Coca‐Cola exposure, supported by SEM evidence of grooves and porosity. This behavior is consistent with the PICN microstructure, where partial dissolution of the feldspathic ceramic network and exposure of the infiltrated polymer phase increase surface heterogeneity [5, 6, 27]. FPMI exhibited an intermediate response, with considerable roughness changes but fewer surface irregularities than PICN according to SEM findings. The limited hydrolytic degradation may be related to its optimized fabrication process, high monomer conversion under high temperature and pressure, and a dense inorganic network with improved resistance to hydrolytic effects [7, 8, 9].

The 3D‐printed resin exhibited the greatest increase in surface roughness (Sa and Sz) after immersion in gastric acid and Coca‐Cola. SEM observations revealed pores and surface irregularities, likely associated with hydrolytic degradation of the Bis‐EMA–based polymer matrix, incomplete polymerization, and limitations inherent to additive manufacturing processes [11, 17, 18, 28, 29]. Although the relatively high ceramic filler content (30–50 wt%) may enhance mechanical performance compared with other printable resins, milled materials still demonstrated superior surface stability [19, 28]. These findings align with previous reports of increased surface roughness, water sorption, and solubility, highlighting the influence of fabrication technique and printing orientation on material performance [12, 17, 29].

### 4.2 Color and Translucency

Color and translucency changes followed a consistent pattern across materials: gastric acid > Coca‐Cola ≫ water. Water storage resulted in minimal Δ*E*₀₀ (< 0.8) and ΔTP₀₀ (< 0.62) values, confirming that changes in acidic groups were primarily chemically driven rather than due to aging alone. Color changes under gastric acid and Coca‐Cola for all materials were clinically acceptable, despite being perceptible (> 0.8). Regarding translucency, EDlithium disilicate was the only material that remained below the perceptibility threshold (< 0.62) in both media, whereas 3D‐printed resin was the only material to exceed the translucency acceptability threshold (> 2.62) after gastric acid exposure.

Lithium disilicate exhibited the greatest translucency stability and the lowest color change, likely owing to its crystalline microstructure and lower susceptibility to acid‐induced structural alteration, consistent with previous studies [2, 16, 26]. In resin‐based materials, translucency is influenced by differences in refractive index between filler and resin matrix, as well as filler size, morphology, and volume fraction [2, 16]. The reduced translucency of PICN may therefore be attributed to refractive index mismatch between its ceramic and polymer networks and its structural characteristics, which increase light scattering, whereas the nanometer‐sized fillers of FPMI, smaller than the wavelength of visible light, limit scattering and enhance light transmission [2, 16]. The highest ΔTP₀₀ values observed for the 3D‐printed resin may be related to increased surface roughness, filler characteristics, monomer type, and polymerization process [16, 17, 18, 19, 26]. Color stability of resin‐ceramic and resin‐based milled materials is affected by water absorption and degradation of the silane‐mediated filler–matrix interface [10, 24]. The higher discoloration of FPMI compared with PICN may be related to differences in microstructure, filler loading and interfacial behavior [2, 10, 16, 24]. Evidence also suggests that Δ*E*₀₀ may be influenced not only by overall resin content, but also by compositional factors, including the chemistry of the organic matrix [7, 24]. In this context, UDMA, used in PICN and FPMI, has been reported to improve color stability compared with Bis‐GMA–based materials due to the absence of hydroxyl groups and lower water sorption [2, 16, 24]. The higher Δ*E*₀₀ observed in the 3D‐printed resin may be associated with acid‐induced surface degradation and enhanced pigment adsorption resulting from the low pH and staining potential of the erosive media [21, 22, 23, 24]. Optical behavior of 3D‐printed resins is strongly influenced by the manufacturing and post‐curing workflow, and suboptimal processing may impair surface integrity and increase residual monomer presence, thereby compromising color stability and translucency [17, 18, 20, 29].

### 4.3 Surface Hardness

Instrumented indentation revealed material‐dependent reductions in Martens hardness, indentation modulus, and elastic index after immersion in gastric acid and Coca‐Cola, following the general pattern: 3D‐printed resin > FPMI≈PICN ≫ lithium disilicate, with minor deviations for nIT% (PICN > FPMI). Lithium disilicate maintained stable mechanical performance due to its load‐bearing crystalline framework, with degradation limited to the glassy phase [3, 4]. The lowest surface hardness values were observed for the 3D‐printed resin, consistent with previous findings showing that aging reduces Martens parameters in 3D‐printed photopolymers due to polymer network relaxation and incomplete interlayer fusion [15]. Reduced hardness is further associated with lower filler content, residual monomers, and anisotropic build structures, which create microstructural heterogeneity and weak interfaces that promote modulus loss and fatigue‐related degradation [13].

These findings should be interpreted in light of certain limitations. Nanoindentation measurements were performed without an integrated optical microscope; therefore, the exact microstructural phase at each indentation site could not be verified. In addition, only one printing orientation and one set of processing parameters were evaluated for the 3D‐printed resin. Future studies should investigate different printing orientations and processing parameters under long‐term, clinically relevant conditions.

## 5 Conclusions

Within the limitations of this study, it was concluded that changes in surface characteristics, optical behavior, and hardness‐related properties differed among the tested restorative materials mainly after acidic immersion, whereas water storage produced only limited changes, with no significant between‐material differences. The 3D‐printed resin showed the least favorable overall response, whereas lithium disilicate showed the greatest resistance to acidic exposure.

The immersion solution affected the magnitude of these changes. Gastric acid produced the most pronounced alterations, followed by Coca‐Cola, while distilled water produced minimal changes.

## Funding

The authors have nothing to report.

## Conflicts of Interest

The authors declare no conflicts of interest related to this study. This research received no external funding. The authors would like to thank Kosmas Tolidis, DDS, PhD, for his valuable scientific guidance and support during the development of this study, as well as Dr. Alexandros Nikolaidis for his assistance with the profilometer measurements and laboratory procedures. The experimental procedures of the scanning electron microscopy (SEM) and Fourier‐transform infrared spectroscopy (FTIR) analyses were performed at the Department of Basic Dental Sciences, Division of Dental Tissues Pathology and Therapeutics, School of Dentistry, Aristotle University of Thessaloniki, Thessaloniki, Greece.

## Data Availability

The data that support the findings of this study are available from the corresponding author upon reasonable request.

## References

[1] T. A. Sulaiman , “Materials in Digital Dentistry: A Review,” Journal of Esthetic and Restorative Dentistry 32 (2020): 171–181. 31943720 10.1111/jerd.12566

[2] M. D. Al Amri , N. Labban , S. Alhijji , et al., “In Vitro Evaluation of Translucency and Color Stability of CAD/CAM Polymer‐Infiltrated Ceramic Materials After Accelerated Aging,” Journal of Prosthodontics 30 (2021): 318–328. 32813300 10.1111/jopr.13239

[3] S. H. Kim , Y. S. Choi , K. H. Kang , and W. Att , “Effects of Thermal and Mechanical Cycling on the Mechanical Strength and Surface Properties of Dental CAD‐CAM Restorative Materials,” Journal of Prosthetic Dentistry 128 (2022): 79–86. 33546857 10.1016/j.prosdent.2020.11.014

[4] S. Chen , C. Y. Kam , Y. H. Lee , X. Bai , Y. Chen , and J. K. H. Tsoi , “Fatigue Behaviour of Fully Crystallised Glass‐Based CAD/CAM Ceramics,” Journal of Dentistry 163 (2025): 106118. 40976277 10.1016/j.jdent.2025.106118

[5] A. Della Bona , P. H. Corazza , and Y. Zhang , “Characterization of a Polymer‐Infiltrated Ceramic‐Network Material,” Dental Materials 30 (2014): 564–569. 24656471 10.1016/j.dental.2014.02.019 PMC4651623

[6] J. F. Nguyen , D. Ruse , A. C. Phan , and M. J. Sadoun , “High‐Temperature‐Pressure Polymerized Resin‐Infiltrated Ceramic Networks,” Journal of Dental Research 93 (2014): 62–67. 24186559 10.1177/0022034513511972 PMC3872849

[7] R. A. Alamoush , N. Silikas , N. A. Salim , S. Al‐Nasrawi , and J. D. Satterthwaite , “Effect of the Composition of CAD/CAM Composite Blocks on Mechanical Properties,” BioMed Research International 2018 (2018): 4893143. 30426009 10.1155/2018/4893143 PMC6218798

[8] K. Barutcigil , A. Dündar , S. G. Batmaz , K. Yıldırım , and Ç. Barutçugil , “Do Resin‐Based Composite CAD/CAM Blocks Release Monomers?,” Clinical Oral Investigations 25 (2021): 329–336. 32488490 10.1007/s00784-020-03377-3

[9] K. Okada , T. Kameya , H. Ishino , and T. Hayakawa , “A Novel Technique for Preparing Dental CAD/CAM Composite Resin Blocks Using the Filler Press and Monomer Infiltration Method,” Dental Materials Journal 33 (2014): 203–209. 24583649 10.4012/dmj.2013-329

[10] C. Lee , S. Yamaguchi , and S. Imazato , “Quantitative Evaluation of the Degradation Amount of the Silane Coupling Layer of CAD/CAM Resin Composites by Water Absorption,” Journal of Prosthodontic Research 67 (2023): 55–61. 34980788 10.2186/jpr.JPR_D_21_00236

[11] N. Güntekin and A. R. Tunçdemir , “Comparison of Volumetric Loss and Surface Roughness of Composite Dental Restorations Obtained by Additive and Subtractive Manufacturing Methods,” Heliyon 10 (2024): e26269. 38390076 10.1016/j.heliyon.2024.e26269 PMC10882017

[12] K. K. Alanazi and A. A. Elkaffas , “Physical Assessment of CAD/CAM and 3D‐Printed Resin‐Based Ceramics Integrating Additive and Subtractive Methods,” Polymers 17 (2025): 2168. 40871116 10.3390/polym17162168 PMC12389419

[13] M. M. Gad , F. A. Al‐Harbi , A. Alrahlah , et al., “A Comparative Study of Strength and Surface Properties of Permanent 3D‐Printed and Milled Composite Resins for Definitive Restorations,” Journal of Prosthodontics 33 (2024): 445–454.

[14] N. Alharbi , D. Wismeijer , and R. B. Osman , “Additive Manufacturing Techniques in Prosthodontics: Where Do We Currently Stand?,” International Journal of Prosthodontics 30 (2017): 474–484. 28750105 10.11607/ijp.5079

[15] M. Reymus and B. Stawarczyk , “In Vitro Study on the Influence of Postpolymerization and Aging on the Martens Parameters of 3D‐Printed Occlusal Devices,” Journal of Prosthetic Dentistry 125 (2021): 817–823. 32444206 10.1016/j.prosdent.2019.12.026

[16] A. Vichi , D. Balestra , N. Scotti , C. Louca , and G. Paolone , “Translucency of CAD/CAM and 3D Printable Composite Materials for Permanent Dental Restorations,” Polymers 15 (2023): 1443. 36987234 10.3390/polym15061443 PMC10053127

[17] S. Baytur and A. A. Diken Turksayar , “Effects of Post‐Polymerization Conditions on Color Properties, Surface Roughness, and Flexural Strength of 3D‐Printed Permanent Resin Material After Thermal Aging,” Journal of Prosthodontics 34 (2025): 298–307. 38102064 10.1111/jopr.13818 PMC11880970

[18] C. K. Scotti , M. M. A. C. Velo , F. A. P. Rizzante , et al., “Physical and Surface Properties of a 3D‐Printed Composite Resin for a Digital Workflow,” Journal of Prosthetic Dentistry 124 (2020): 614.e1–614.e5. 10.1016/j.prosdent.2020.03.029 32636072

[19] A. C. A. Zattera , F. A. Morganti , G. S. Balbinot , G. Souza Balbinot , A. Della Bona , and F. M. Collares , “The Influence of Filler Load in 3D Printing Resin‐Based Composites,” Dental Materials 40 (2024): 1041–1046. 38763819 10.1016/j.dental.2024.05.016

[20] E. J. A. Lalvay , F. G. de Carvalho , A. R. da Silva , F. A. C. Morganti , F. M. Collares , and E. A. Münchow , “3D‐Printed Resins Intended for Long‐Term Restoration vs. Conventionally Used Materials: A Systematic Review and Network Meta‐Analysis of in Vitro Studies,” Journal of Dentistry 167 (2026): 106531. 41628707 10.1016/j.jdent.2026.106531

[21] T. Donovan , “Dental Erosion,” Journal of Esthetic and Restorative Dentistry 21 (2009): 359–364. 20002921 10.1111/j.1708-8240.2009.00291.x

[22] A. Elraggal , R. R. Afifi , R. A. Alamoush , et al., “Effect of Acidic Media on Flexural Strength and Fatigue of CAD‐CAM Dental Materials,” Dental Materials 39 (2023): 57–69. 36496258 10.1016/j.dental.2022.11.019

[23] M. Alnasser , M. Finkelman , A. Papathanasiou , M. Suzuki , R. Ghaffari , and A. Ali , “Effect of Acidic pH on Surface Roughness of Esthetic Dental Materials,” Journal of Prosthetic Dentistry 122 (2019): 567.e1–567.e8. 10.1016/j.prosdent.2019.08.022 31699448

[24] S. B. Patel , V. V. Gordan , A. A. Barrett , and C. Shen , “The Effect of Surface Finishing and Storage Solutions on the Color Stability of Resin‐Based Composites,” Journal of the American Dental Association (1939) 135 (2004): 587–594. 15202750 10.14219/jada.archive.2004.0246

[25] A. Gil‐Pozo , D. Astudillo‐Rubio , Á. Ferrando Cascales , et al., “Effect of Gastric Acids on the Mechanical Properties of Conventional and CAD‐CAM Resin Composites—An in Vitro Study,” Journal of the Mechanical Behavior of Biomedical Materials 155 (2024): 106565. 38718723 10.1016/j.jmbbm.2024.106565

[26] M. E. M. Cruz , R. Simões , S. B. Martins , F. Z. Trindade , L. N. Dovigo , and R. G. Fonseca , “Influence of Simulated Gastric Juice on Surface Characteristics of CAD‐CAM Monolithic Materials,” Journal of Prosthetic Dentistry 123 (2020): 483–490. 31383520 10.1016/j.prosdent.2019.04.018

[27] P. Mourouzis , A. Tsiveli , P. Tsetseli , C. Gogos , and K. Tolidis , “Impact of Erosion and Aging Simulation on Chairside Materials,” Microscopy Research and Technique 86 (2023): 943–954. 37218339 10.1002/jemt.24361

[28] Formlabs , Permanent Crown Resin: Instructions for Use (Formlabs, 2021).

[29] C. Espinar , A. D. Bona , M. M. Pérez , M. Tejada‐Casado , and R. Pulgar , “The Influence of Printing Angle on Color and Translucency of 3D Printed Resins for Dental Restorations,” Dental Materials 39 (2023): 410–417. 36914433 10.1016/j.dental.2023.03.011

[30] C. M. L. Bollen , P. Lambrechts , and M. Quirynen , “Comparison of Surface Roughness of Oral Hard Materials to the Threshold Surface Roughness for Bacterial Plaque Retention: A Review of the Literature,” Dental Materials 13 (1997): 258–269. 11696906 10.1016/s0109-5641(97)80038-3

[31] M. Salas , C. Lucena , L. J. Herrera , A. Yebra , A. Della Bona , and M. M. Pérez , “Translucency Thresholds for Dental Materials,” Dental Materials 34 (2018): 1168–1174. 29764698 10.1016/j.dental.2018.05.001

[32] International Commission on Illumination , Colorimetry, 3rd ed. (Commission Internationale de l'Eclairage, 2004).

[33] R. D. Paravina , M. M. Pérez , and R. Ghinea , “Acceptability and Perceptibility Thresholds in Dentistry: A Comprehensive Review of Clinical and Research Applications,” Journal of Esthetic and Restorative Dentistry 31 (2019): 103–112. 30891913 10.1111/jerd.12465

[34] International Organization for Standardization , Dentistry—Guidance on Colour Measurement. ISO/TR 28642:2016 (ISO, 2016).

[35] W. C. Oliver and G. M. Pharr , “Measurement of Hardness and Elastic Modulus by Instrumented Indentation: Advances in Understanding and Refinements to Methodology,” Journal of Materials Research 19 (2004): 3–20.

